# When is the Best Time for the Second Antiplatelet Agent in Non-St Elevation Acute Coronary Syndrome?

**DOI:** 10.5935/abc.20160042

**Published:** 2016-03

**Authors:** Pedro Gabriel Melo de Barros e Silva, Henrique Barbosa Ribeiro, Antônio Claudio do Amaral Baruzzi, Expedito Eustáquio Ribeiro da Silva

**Affiliations:** 1 Hospital TotalCor, São Paulo, SP - Brazil; 2 Brazilian Clinical Research Institute (BCRI), São Paulo, SP - Brazil; 3 Instituto do Coração - Hospital das Clínicas - Faculdade de Medicina da Universidade de São Paulo, São Paulo, SP- Brazil

**Keywords:** Acute Coronary Syndrome / therapy, Platelet Aggregation Inhibitors / administration & dosage, Aspirin / administration & dosage, Percutaneous Coronary Intervention

## Abstract

Dual antiplatelet therapy is a well-established treatment in patients with non-ST elevation acute coronary syndrome (NSTE-ACS), with class I of recommendation (level of evidence A) in current national and international guidelines. Nonetheless, these guidelines are not precise or consensual regarding the best time to start the second antiplatelet agent. The evidences are conflicting, and after more than a decade using clopidogrel in this scenario, benefits from the routine pretreatment, i.e. without knowing the coronary anatomy, with dual antiplatelet therapy remain uncertain. The recommendation for the upfront treatment with clopidogrel in NSTE-ACS is based on the reduction of non-fatal events in studies that used the conservative strategy with eventual invasive stratification, after many days of the acute event. This approach is different from the current management of these patients, considering the established benefits from the early invasive strategy, especially in moderate to high-risk patients. The only randomized study to date that specifically tested the pretreatment in NSTE-ACS in the context of early invasive strategy, used prasugrel, and it did not show any benefit in reducing ischemic events with pretreatment. On the contrary, its administration increased the risk of bleeding events. This study has brought the pretreatment again into discussion, and led to changes in recent guidelines of the American and European cardiology societies. In this paper, the authors review the main evidence of the pretreatment with dual antiplatelet therapy in NSTE-ACS.

## Introduction

Large clinical studies have demonstrated a beneficial effect of the antiplatelet therapy using the combination of a P2Y12 receptor and acetylsalicylic acid (ASA) in non-ST segment elevation acute coronary syndrome (NSTE-ACS).^1-3^ This association has been widely used in the last decade with successful application in real world.^4,5^ Nevertheless, after ten years of dual antiplatelet therapy (DAPT) in NSTE-ACS, some gaps still exist. One of the controversial practical issue relates to the timing for starting the second antiplatelet agent to inhibit P2Y12 receptor (adenosine diphosphate - ADP - pathway). It is still unclear whether the pretreatment is really beneficial compared to the introduction of the second antiplatelet drug after the knowledge of the coronary anatomy.^6-8^

The present article presents a brief discussion about the indication of DAPT in NSTE-ACS, and evaluates the benefits of the early invasive strategy and the main evidence of the best time for the use of antiplatelet therapy in NSTE-ACS.

### Main studies on dual antiplatelet therapy

The three major studies^1-3^ that demonstrated the clinical benefit of the DAPT were the CURE (Clopidogrel in Unstable Angina to Prevent Recurrent Events) with clopidogrel, the TRITON-TIMI^38^ (Trial to Assess Improvement in Therapeutic Outcomes by Optimizing Platelet Inhibition with Prasugrel-Thrombolysis in Myocardial Infarction^38^ with prasugrel, and the PLATO (Platelet Inhibition and Patient Outcomes) with ticagrelor. While the first study included only patients with NSTE-ACS, the others also involved patients with ST elevation myocardial infarction (STEMI). Another difference was that while in the CURE study, the second antiplatelet agent (in this case, clopidogrel) was compared to placebo, in the TRITON and PLATO studies, the new antiplatelet was compared with clopidogrel. Considering the inclusion criteria (which included the presence of ST-segment deviation in the electrocardiogram or increased markers for myocardial necrosis), it becomes evident that the study population was composed by NSTE-ACS patients with a greater risk, being most of them composed of non-ST elevation myocardial infarction (NSTEMI). With respect to lower-risk patients, they were included in the initial phase of the CURE study, represented by patients aged over 60 years, with no changes in the electrocardiogram, but with previous history of coronary disease. After a review of the event rates in the first 3,000 patients, however, the study committee recommended that only those patients with changes in the electrocardiogram or myocardial necrosis markers should be included, since therapeutic benefit could not be demonstrated in less severe cases. This preliminary analysis demonstrated that the routine use of a second antiplatelet agent (clopidogrel) in lower-risk patients had little or no benefit as compared with placebo. The TRITON and PLATO clinical trials did not include unstable angina patients without ST deviation. This information should be considered in the initial care of patients with chest pain and no changes in ST-segment or markers for myocardial necrosis, since even though the evidence of these studies^1-3^ may be applicable to lower-risk patients, in general, the lower the risk, the lower the absolute benefit, and a more individualized therapy should be selected.

The primary and safety outcomes were similar among the three studies. From the critical analysis of these studies, the following conclusions can be drawn: (1) the DAPT should be routinely performed in patients with NSTE-ACS, especially when they have positive myocardial necrosis markers and/or st-segment changes on the electrocardiogram; (2) with respect to the primary outcome (study question) - composed by cardiovascular death, acute myocardial infarction (AMI) and stroke - clopidogrel was superior to placebo, with a number needed to treat (NNT) of 48, and the two new antiplatelet agents (prasugrel and ticagrelor) were superior to clopidogrel (both with a NNT around 50); (3) the risk of bleeding was higher with clopidogrel than with placebo, with a number needed to harm (NNH) of 100, whereas the new antiplatelet agents increased the risk of major bleeding not related to surgery (both with a NNH near 150).

Prasugrel was superior to clopidrogrel in patients with scheduled percutaneous coronary intervention (PCI) in the TRITON study, whereas in the PLATO trial, ticagrelor was tested in three types of treatment (medical only, PCI or surgical). There is no direct comparison between prasugrel and ticagrelor that would suggest a better option between them. Both are superior to clopidogrel in patients undergoing PCI in ACS. Secondary outcomes and subgroup analysis in the TRITON and PLATO studies may help in the decision of the best therapy for each patient. Also, drug-related issues, including costs, posology and adverse effects may be useful in the therapeutic decision making.

### Early invasive strategy and the concept of pre-treatment

Several studies have compared the early invasive strategy vs. conservative or selective invasive strategy in NSTE-ACS.^9^ Different concepts of these strategies and different adjuvant therapies explain, in part, discrepancies in the results. However, studies using more contemporary concepts regarding adjunctive treatments (ASA, thienopyridines and/or glycoprotein iib/iiia inhibitors) and use of stents in patients undergoing PCI have shown greater benefit from early invasive strategy (coronary angiography and sequential revascularization). A meta-analysis conducted in 2006,^9^ including seven studies and 8,375 patients showed a significant reduction of 25% in all-cause mortality (4.9% vs. 6.5%; p = 0.001), and of 17% in non-fatal AMI (7.6% vs. 9.1%; p = 0.012) within two years of follow-up, with no increase of adverse effects.

In light of the benefits of early invasive strategy with revascularization in NSTE-ACS, new studies have tested earlier strategies of stratification. A recent meta-analysis^10^ involving 4,013 patients compared the early stratification within 1 and 14 hours with the strategy between 20.8 e 86 hours. No difference in the endpoints - death and non-fatal infarction - was observed between the interventions. However, the early strategy was associated with a lower risk of recurrent ischemia, shorter hospitalization, and a trend of lower risk of bleeding and the composite of death, AMI and stroke. Although this metanalysis has not stratified the patients according to the risk, the TIMACS^11^(Timing of Intervention in Acute Coronary Syndrome) study showed a 35% reduction of death, infarction and stroke in the high-risk subgroup assigned to invasive stratification within 24 hours. The positive results of these studies, showing the safety and potential benefit of the invasive stratification within 24 hours have led to changes in the recommendations of recent international guidelines.^12,13^

### Time for the second antiplatelet agent

In the three main clinical trials that evaluated the efficacy of the three oral antiplatelet agents that have been approved to be used in combination with ASA (clopidogrel, prasugrel and ticagrelor), different approaches were used to administer the second antiplatelet agent. In the CURE^1^ and PLATO,^3^ studies, it was started during patients' recruitment, at 14 hours and 11 hours (median) from onset of pain in the CURE and PLATO study, respectively (and mean of 5 hours from hospital admission in the PLATO study). In the TRITON study,^2^ patients received the second antiplatelet drug in the catheterization laboratory, similar to the CHAMPION PHOENIX (Cangrelor versus standard therapy to acHieve optimal Management of Platelet InhibitiON PHOENIX trial).^14^ In this study,^14^ the authors used cangrelor (not approved in Brazil yet), and they have chosen not to use the pre-treatment since this is a widely used practice in many centers.^15^ These studies^1-3,14^ have not tested the pretreatment hypothesis, but rather evaluated the benefit (or not) of the second antiplatelet agent in comparison with placebo (the CURE study) or clopidogrel (PLATO and TRITON studies). An important aspect is that, in the CURE study, only 43% of patients underwent angiography and 21% PCI; the procedures were performed 10 days (median) from the acute event, and one third of them were conducted after hospital discharge. This is explained by the fact that the CURE study included particularly centers where the invasive stratification was not performed. Thus, the approach of this study is not suitable for the current concept of pretreatment in NSTE-ACS,^7^ which includes early invasive strategy, especially in higher-risk cases.

In NSTE-ACS, the concept of pretreatment should be applied to the therapy used before the coronary angiography in patients undergoing early invasive approach. The discussion about pretreatment does not apply to those cases in which a conservative approach has been initially chosen, since generally, there is no decision on whether or not (and when) a coronary angiography will be performed.

The main reasons in favor of or against the pretreatment, are presented in chart 1, and will be fully described below.

**Chart 1:** Main arguments in favor and against the pretreatment in ACS

**Reasons in favor of the pretreatment**	**Reasons against the pretreatment**
Biological plausibility for reduction of ischemic events	Biological plausibility for increased bleeding risk
The benefits of the DAPT were consistent with all treatments, including surgical revascularization; a minority of patients with NSTE-ACS undergo myocardial revascularization in the first week	The main studies on DAPT in ACS have not been designed to evaluate pretreatment. There is an increased risk of surgical bleeding during the first days after the use of DAPT
A meta-analysis proved a reduction in the non-fatal ischemic events	Studies showing a reduction in the non-fatal ischemic events used selective invasive strategy, and such effect was not reproduced in similar studies on early catheterization
There is no class effect, and different characteristics have been found between prasugrel and ticagrelor	The only study that properly tested the pretreatment (ACCOAST) failed to prove the benefit of this hypothesis, and showed the risk of this strategy
The CURE study showed a benefit in the first 24 hours	Evidence have suggested that the early catheterization may counterbalance the benefit of the pretreatment

NSTE-ACS: non-STsegment elevation acute coronary syndrome; DAPT dual antiplatelet therapy; ACS: acute coronary syndrome.

### Biological plausibility

This is one of the most common explanations to justify the need to rapidly start the second antiplatelet drug, even before evaluating the anatomy of the coronary arteries. Considering that NSTE-ACS results from platelet-rich thrombus formation, and that the DAPT shows clinical benefit, it is expected that the earlier the administration of the second antiplatelet agent, the better for the patient. Besides being a reasonable decision, this practice also brings comfort to the physician, since an early intervention seems to avoid complications related to acute thrombotic events. However, the routine use of pretreatment may also pose risks, since the same potentially protective antiplatelet effect could also increase the bleeding risk, especially considering the most potent antiplatelet drugs, when associated with other antithrombotic agents or during invasive interventions. In addition to this potential risk, nearly 10% of patients with NSTE-ACS would not benefit from the upfront DAPT, since these patients do not have angiographic features of obstructive coronary disease, according to data from the CRUSADE (Can Rapid Risk Stratification of Unstable Angina Patients Suppress Adverse Outcomes with Early Implementation of the ACC/AHA Guidelines). This percentage reaches 15% among women.^16^

Finally, besides the theoretical uncertainties about the net benefit (ischemia *vs*. bleeding), one should take into account that there are many examples of practices in the scientific literature based on biological plausibility that do not show any clinical benefit, and may rather be harmful when tested with rigorous methodology.^17^ Therefore, despite the stronger hypothesis of the benefit from the pretreatment, the evaluation of its clinical effect is still needed. Also, whether a more potent antiplatelet agent prior to early catheterization would safely reduce ischemia should also be assessed.

### Surgical risk

The potential harm of the pretreatment is even more plausible in patients undergoing surgical treatment, especially within less than one week after the P2Y12 inhibitor is discontinued. In the CURE study, 16.5% of patients underwent myocardial revascularization surgery; the median time from randomization to the surgery was 26 days, and 12 days among hospitalized patients.^18^ An argument in favor of the pretreatment is that, even in a specific analysis of the surgical patients, the combined endpoint of cardiovascular death, STEMI or stroke was lower for those patients receiving clopidogrel, although this did not reach statistical significance vs. placebo (relative risk - RR: 0.82; 95% confidence interval - 95%CI 0.58-1.16). However, the comparison of the major bleeding outcomes were also consistent with the main study, indicating a higher risk of bleeding in such patients undergoing the pretreatment with clopidogrel vs. placebo but without statistical significant difference (RR: 1.27; 95%CI 0.96-1.69; p=0.095). Although post-hoc observations of other clinical trials have not found increased major bleeding rates,^19^ observational studies have demonstrated a significant increase of transfusion and reoperation in patients that received clopidogrel up to 5 days before surgical myocardial revascularization. This was corroborated by a systematic review and meta-analysis of observational data showing a 30% increase in mortality.^20^

On the other hand, only 10% to 20% of patients with NSTE-ACS are treated with surgical revascularization^21^ and many of them after 5 days of the initial hospitalization. Thus, the potential benefit in a large group of patients (which will not undergo surgical myocardial revascularization), may suggest that the risk of pretreatment would not outweigh the benefits. However, neither the benefits nor the risks have been defined in early stratification, and the definition of the best moment for DAPT should be based on adequate studies.

### Studies that tested the pretreatment hypothesis

Table 1 depicts a summary of the main studies that evaluated the pretreatment hypothesis in NSTE-ACS and in the following paragraphs are additional aspects of two of them.

**Table 1:** Characteristics of clinical trials that evaluated the use of pretreatment with thienopyridines in patients with non-ST segment elevation acute coronary syndrome (NSTE-ACS)

**Study**	**Type of study**	**NSTE-ACS n (%)**	**Patients undergoing PCI n (%)**	**Pretreatment**	**Loading dose in the group of patients without pretreatment**	**Main study outcome (composite)**	**Safety outcome**	**NNT/ NNH**
**Clopidogrel**								
CREDO	Randomized, clinical trial	1,407/2,116 (66.5)	1,820/2,116 (86.0)	300 mg of loading dose 3-24 hours before PCI (mean of 9.8 hours)	Without loading dose; patients received clopidogrel 75 mg during 28 days	Death, AMI, UTVR (per protocol analysis)	TIMI major and minor bleeding	*/*
CURE	Randomized, clinical trial	12,562/12,562 (100)	2,663/12,562 (21.2)	300 mg of loading dose (median of 10 days pre-PCI), followed by 75 mg for 3-12 months	Without loading dose; patients with PCI received clopidogrel 75 mg during 28 days	Cardiovascular death, AMI, stroke	Major bleeding	48/100
PCI-CURE	Subgroup of a randomized, clinical trial	2,658/2,658 (100)	2,658/2,658 (100)	300 mg of loading dose (median of 10 days pre-PCI), followed by 75 mg for 3-12 months	Without loading dose; patients received clopidogrel 75 mg during 28 days	Cardiovascular death, AMI, UTVR	Major bleeding	53/*
ACUITY	Subgroup of a randomized, clinical trial	7,523/7,523 (100)	4,243/7,523 (56.4)	Subgroup ≥ 300 mg of loading dose	Subgroup ≥ 300 mg of loading dose post-PCI < 2 hours	Cardiovascular death, AMI, UTVR	Major bleeding	*/*
ACUITY-PCI	Non randomized, prespecified analysis of a subgroup of a clinical trial	5,039/5,039 (100)	5,039/5,039 (100)	Subgroup ≥ 300 mg of loading dose	Subgroup ≥ 300 mg of loading dose post-PCI < 2 hours	Cardiovascular death, AMI, UTVR	Major bleeding	*/*
**Prasugrel**								
ACCOAST	Randomized, clinical trial	4,033/4,033 (100)	2,770/4,033 (68.7)	30 mg of Prasugrel 30 mg 2-48 hours before angiography (median of 4.4 hours), followed by 30 mg prior to PCI	60 mg of Prasugrel prior to PCI (after angiography)	Cardiovascular death, AMI, UTVR, stroke. Use of glycoprotein iib/iiia inhibitors	TIMI major and minor bleeding	*/83

* No statistically significant difference was observed between the pretreated group and the group without pretreatment. NNT/NNH: number needed to treat / number needed to cause harm; PCI: percutaneous coronary intervention; AMI: acute myocardial infarction; UTVR: urgent target-vessel revascularization; TIMI: thrombolysis in myocardial infarction.

### PCI-CURE

The PCI-CURE^22^ study assessed patients who have undergone PCI in the CURE study (21% of initial sample). After PCI, more than 80% of patients received open-label thienopyridine for 4 weeks, after which they received the study drug again for a mean of 8 months. As compared with placebo, the authors found a benefit from the use of clopidogrel (for a median of 10 days) before PCI, with reduction of the composite endpoint of lower cardiovascular death, myocardial infarction, or urgent target-vessel revascularization (UTVR) within 30 days of PCI (4.5% *vs*. 6.4%; p = 0.03). There was no reduction in cardiovascular death alone, but there was a reduction in cardiovascular death and myocardial infarction at 30 days, although this benefit was not statistically significant at 48 hours or 7 days of follow-up.

### CREDO

This study^23^ was designed to specifically evaluate pretreatment with clopidogrel, and included more than half of patients with ACS. The loading dose of clopidogrel was initiated at 3-24 hours (mean of 9.8 hours) before PCI. No significant reduction was found with regard to ischemic events (death, AMI, and UTVR) at 28 days of pretreatment (6.8% vs. 8.3%; p = 0.23), and there was a trend for increased major bleeding events (8.8% vs. 6.7%; p = 0.07).

Considering the above mentioned studies, the PCI-CURE study was the main investigation that demonstrated a benefit from the therapy with clopidogrel before catheterization in NSTE-ACS. However, considering that both angiography and PCI were rarely indicated in this study, the PCI-CURE results may not be applicable to the current pretreatment concept, since this therapeutic regimen is based on performing coronary angiography routinely. Studies with appropriate methodology (prospective and randomized) to answer this question, such as the CREDO study, did not corroborate the benefit of clopidogrel pretreatment. Despite this fact, a joint analysis of these studies in a systematic review and meta-analyses would increase the power of this investigation and minimize the probability of type II error.

### Meta-analyses

In 2012, a meta-analysis^24^ that included both observational studies and clinical trial showed that DAPT with clopidogrel and ASA before angioplasty did not reduce mortality, but reduced the risk for major cardiovascular events. This meta-analysis included not only studies with different methodologies, but also studies on different clinical conditions (stable coronary disease, NSTE-ACS and STEMI) and distinct stages of angiographic evaluation. The main analysis, which included only clinical trials, showed that the pretreatment with clopidogrel was not associated with lower mortality (1.54% vs. 1.97%; p = 0.17), but did associate with lower risk for cardiovascular events (9.83% vs. 12.35%; p = 0.001). Likewise, no significant association was found between the pretreatment and higher major bleeding rates (3.57% vs. 3.08%; p = 0.18). The results were heterogeneous according to the clinical presentation: in patients with stable coronary disease, no reduction of ischemic events was observed, and there was a trend towards a higher risk of bleeding; while in the context of NSTE ACS there were lower cardiovascular events (13.91% vs. 17.19%; p = 0.002) and a trend for more bleeding risk (Odds Ratio - OR: 1.28; p = 0.07).

In 2013, a new systematic review and meta-analysis on patients undergoing PCI^25^ was conducted. These patients are the ones who may benefit the most from pre-catheterization DAPT. Nonehteless, the authors found no clinical benefit and a potential risk of bleeding in the pretreatment group.

**Figure 1:**
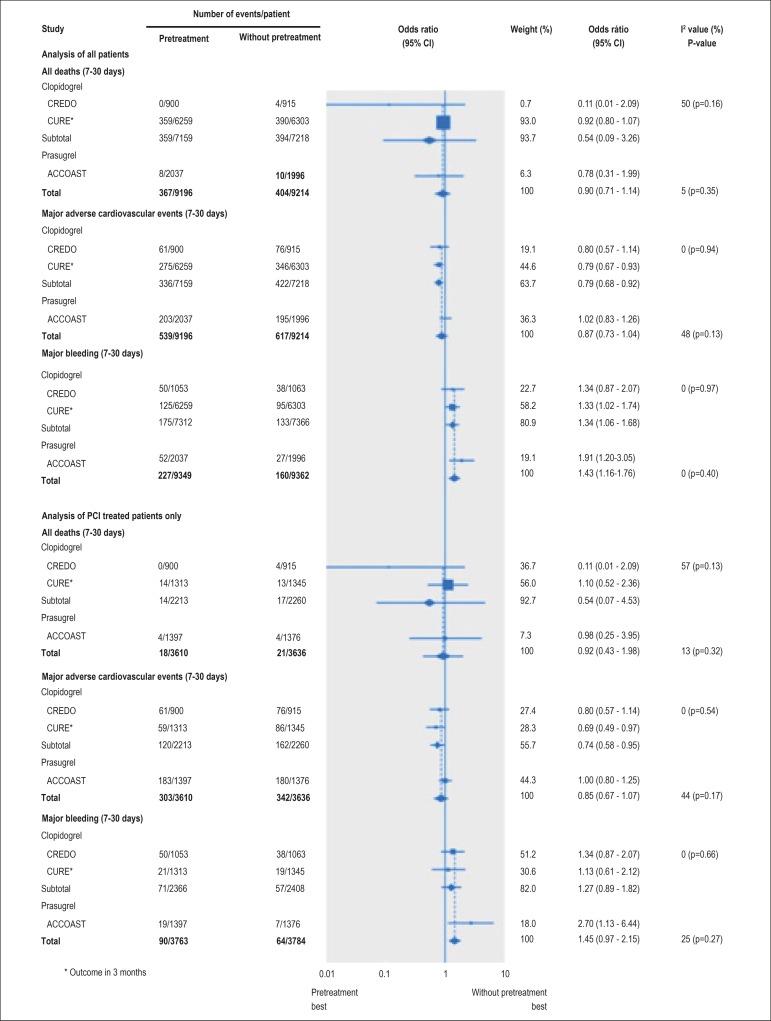
Forest-Plot of the clinical trials included in the meta-analysis on pretreatment with thienopyridines in non-ST segment elevation acute coronary syndrome.

In 2014, a systematic review and meta-analysis on pretreatment in NSTE-ACS^26^ was published. The study included 32,383 patients, 18,711 of whom from randomized, controlled studies. Fifty-five percent of the patients underwent PCI. Only studies on thienopyridine were included, since there were no investigations on other antiplatelet agents in NSTE-ACS. Although the pretreatment did not significantly affect the mortality rate, a significant increase of 30-45% in major bleeding events was detected. These results were consistent with the assessment of all patients as well as in the PCI subgroup. A lower cardiovascular event rate in the pretreatment group was identified in the CURE study. However, surprisingly, no significant difference in the cardiovascular event rate was found in the group of patients undergoing PCI (condition in which a higher benefit from the pretreatment would be expected). Figure 1 depicts the forest plot of all clinical trials included in this meta-analysis. The results of the meta-analysis do not support the pretreatment strategy as a routine practice in NSTE-ACS, due to the lack of a favorable risk-benefit balance, especially with respect to the absence of a benefit in cardiovascular events among contemporary studies.

### ACCOAST study and the class effect

As previously mentioned, most of the evidence of pretreatment with clopidogrel in NSTE-ACS comes from studies about other practices different from the early invasive strategy (which is the currently recommended approach). The only clinical trial that tested the hypothesis of the pretreatment using early invasive stratification was the ACCOAST (Comparison of Prasugrel at the Time of Percutaneous Coronary Intervention or as Pretreatment at the Time of Diagnosis in Patients with Non-ST Elevation Myocardial Infarction) trial,^27^ which used prasugrel as the second antiplatelet agent. The study included 4,033 patients with NSTEMI, who were randomized, in a double-blind manner, to receive 30 mg of prasugrel or placebo (control group) before coronary angiography was performed. After this procedure, 69% of patients underwent PCI and received an additional 30 mg or 60 mg (control group) of prasugrel. No difference in the primary composite endpoint (death from cardiovascular causes, AMI, stroke, urgent revascularization, or use of glycoprotein IIb/IIIa inhibitor) was found between the groups within 7 days (*Hazard Ratio* - HR: 1.02; p = 0.81), or 30 days. On the other hand, the frequency of major bleeding episodes was twice higher in the pretreatment group at day 7 and day 30 after randomization (p < 0.01). The study was interrupted early due to excess bleeding complications and lack of clinical benefit. Both the lack of efficacy and the safety issues of the pretreatment were consistent throughout the analyses of subgroups. In patients undergoing PCI, there was a three-time higher rate of TIMI major bleeding, and six-time higher rate of life-threatening bleeding not related to myocardial revascularization.^27,28^

In light of this study, the best time for administration of the second antiplatelet agent has been questioned again in the contemporary practice of early invasive stratification, especially involving more recent drugs, and led to changes in recent guidelines.^12,13^ Considering that routine angiography is performed in many centers, and that recent antiplatelet drugs have high potency and very fast action, a possible benefit of achieving an antiplatelet effect before the angiography is performed may seem irrelevant. The possibility that a higher antiplatelet action would be sufficient to minimize the ischemic events was also questioned in this study, since the pharmacodynamic analysis revealed a lower platelet aggregation in the pretreatment group than in the controls at the time of the procedure. Therefore, although the pretreatment led to a higher antiplatelet action, such effect was not sufficient to reduce clinical endpoints related to myocardial ischemia, but was associated with higher bleeding complications rate. At 2 hours after the second loading dose, the antiplatelet activity was similar in the two groups. In addition, analysis of patients undergoing PCI showed that, although the identification of thrombus in the angiography was an independent predictor of a three-time higher rate of events when compared to patients without thrombus, no difference was found in the presence of thrombus between the pretreatment group and controls. Finally, there was no reduction in stent thrombosis post-PCI, and the incidence of ischemic events was the same in both therapeutic strategies.^28^

A rationale for the use of pretreatment even after the ACCOAST trial is based on the absence of class effect among the antiplatelet agents. Differently from thienopyridines, ticagrelor does not require metabolic activation, and acts in the ADP pathway by reversible inhibition of the P2Y12 receptor.^29^ Besides, other effects via adenosine may explain differences between the classes of antiplatelet drugs.^30^ So far, there is no randomized clinical trial that compared the use of ticagrelor before and after knowing the coronary anatomy in NSTE-ACS. The ATLANTIC^31^ study evaluated the early introduction of ticagrelor in STEMI, by comparing the administration of a loading dose in the ambulance *vs* in the catheterization laboratory. Although the STEMI patients have the greatest potential to benefit from the pretreatment,^24^ the ATLANTIC study did not show any benefit from this strategy in the coprimary endpoints. Although the results of the ATLANTIC study may raise doubts about the real benefits of the pretreatment, ticagrelor was shown to be safe in relation to bleeding events in primary angioplasty in STEMI. Also, it suggested a potential benefit related to lower stent thrombosis rate (secondary outcome), which, in general, supports the practice of early DAPT in STEMI but does not change the question regarding pretreatment in NSTE-ACS.

### Pretreatment and the moment for the coronary angiography

In the ACCOAST trial, the time elapsed from the loading dose of prasugrel to angiography was 4.3 hours; it was a relatively short period, but longer than other recent studies.^3,32,33^ As compared to the clinical practice, this time would be longer, since it did not include the time required for diagnosis, presentation of the informed consent form and randomization, which occurred before the loading dose administration. No benefit, however, was observed from the pretreatment in reducing ischemic events even in patients undergoing PCI above the median time of 4.3 hours in the ACCOAST trial, in which a maximum time of 48 hours was tolerated for stratification. Since clinical trials may not reflect the real world, any therapy found effective in these studies should be assessed in clinical practice. In this context, the recent TRANSLATE-ACS (Treatment with ADP receptor Inhibitors: Longitudinal Assessment of Treatment Patterns and Events after Acute Coronary Syndrome) study,^15^ which evaluated current practices of adjunct therapy, showed that pretreatment with both clopidogrel and prasugrel was associated with a similar risk of intrahospital major cardiovascular events as compared with the treatment after knowing the coronary anatomy. Nevertheless, in the TRANSLATE-ACS study^15^, there was no evidence of differences in bleeding rates between pretreatment and control (without pretreatment).

**Figure 2:**
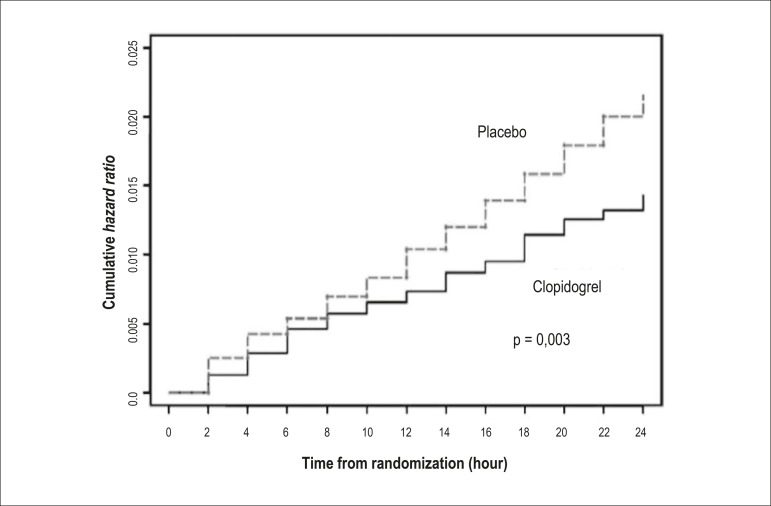
Event curve (composite endpoint of death due to cardiovascular disease, stroke, acute myocardial infarction and major ischemia) in clopidogrel group vs. placebo group in the first 24 hours in the CURE study^1^. Relative risk of 0.66; p < 0.01.

**Figure 3:**
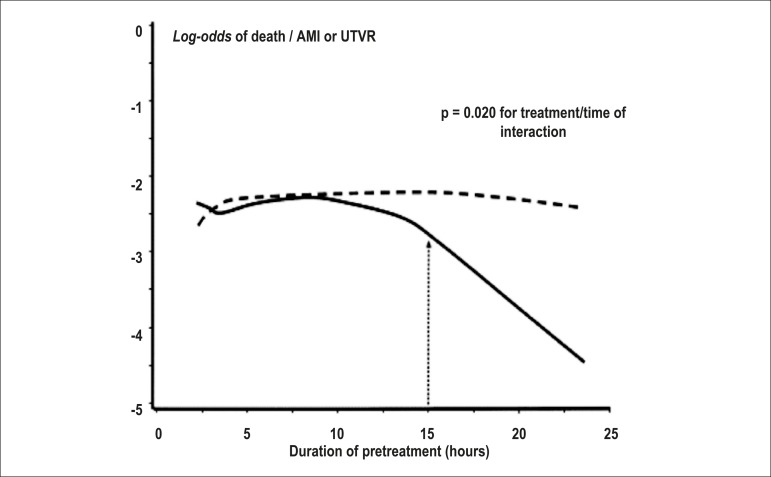
Analysis of the benefit (death, infarction or urgent target-vessel revascularization) of pretreatment, by the time from drug administration to catheterization in the CREDO study.^23,34^ Dotted line indicates the placebo group (without pretreatment). A significant reduction in events at 15 hours from the pretreatment with clopidogrel is observed. AMI: acute myocardial infarction; UTVR: urgent target-vessel revascularization

In light of these questions, indirect data may be interesting to define a maximum tolerable period without pretreatment. Time analysis of the CURE^1^ study demonstrated a reduction in the composite endpoint of death due to coronary disease, stroke and AMI in the first 30 days, and in these three conditions when associated with ischemia in the first 24 hours (Figure 2). Subanalysis of CREDO^23,34^ suggested that a loading dose of clopidogrel at least 6 hours before PCI may be beneficial (38.6% reduction in RR; p = 0.051), although the cutoff in hours best associated with differences in favor of the pretreatment was 15 hours (Figure 3).^34^

The hypothesis of a benefit in the pretreatment group at a time longer than 6 hours, as suggested in the CREDO study, was assessed in two clinical trials with appropriate methodology, although these studies did not include patients with ACS. The PRAGUE-8^35^ (Clopidogrel Only Before Percutaneous Coronary Intervention or Before Every Coronarography?) and ARMYDA-5 (Antiplatelet therapy for Reduction of Myocardial Damage during Angioplasty)^36^ studies evaluated patients with stable disease, using a loading dose of 600mg of clopidogrel > 6 hours before the PCI, and no reduction of ischemic events was observed. In the PRAGUE-8 study, this result was associated with excessive bleeding.

### Recommendations of guidelines

Table 2 summarizes changes in the recommendations about pretreatment in recent guidelines of the main international cardiology organizations.^12,13,37-44^

**Table 2:** Summary of changes in the guidelines' recommendations through the time

**Cardiology society**	**Year**	**Recommendation of pretreatment**
American Cardiology of Cardiology/ American Heart Association	2007^38^	The pretreatment is recommended, although the guideline also present the following sentence: "initiation of clopidogrel may be deferred until a revascularization decision is made"
	2012^41^	Patients diagnosed with moderate or high-risk NSTE-ACS should receive dual antiplatelet therapy (pre-catheterization)
	2014^12^	There is no clear recommendation for DAPT before knowing the coronary anatomy. It recommends a loading with P2Y12 inhibitor in patients who will undergoing PCI with stenting
	2007^39^	"Postponing clopidogrel to after angiography cannot be recommended"
	2010^40^	
	2011^43^	A P2Y12 inhibitor should be used as soon as possible
European Society of Cardiology	2014^44^	Pretreatment with prasugrel in patients in whom coronary anatomy is not known: class of recommendation III, level of evidence B*
	2015^13^	There is a specific session to discuss the best moment for P2Y12 administration, which highlights the controversy of the subject. Since there is no appropriate investigation on clopidogrel and ticagrelor, the guidelines do not specify any recommendation (in favor or against) on pretreatment in early invasive strategy, and do not recommend pretreatment with prasugrel. In conservative approach, P2Y12 inhibitor should be initiated (preferentially ticagrelor) as soon as the diagnosis is confirmed.
Sociedade Brasileira de Cardiologia	2013^37^	In both, there is no formal recommendation about the best moment for the second antiplatelet agent, and prasugrel is recommended only after the coronary anatomy is known
	2014^42^	

* With respect to clopidogrel and ticagrelor in NSTE-ACS, these guidelines do not make any specific recommendation, but bring a discussion about related evidence, and reinforce that the pretreatment with ticagrelor had not been tested yet. NSTE-ACS: non-ST-segment-elevation acute coronary syndrome; DAPT: dual antiplatelet therapy; PCI: percutaneous coronary intervention.

## Conclusions

Considering the currently available evidence, although biologically attractive and intuitive, the benefit from the pretreatment with DAPT has not been proved in randomized, prospective studies, and diverging opinions about the best approach have been lingering in the medical community.^7,8^ In centers in which early invasive stratification is not performed in NSTE-ACS, evidence from the PCI-CURE study should be applied and the P2Y12 should be early administered. On the other hand, in centers in which the early invasive stratification is performed, the evidence and recommendation of current guidelines provide us with two therapeutic options - pretreat or not pretreat, according to the choice of the second antiplatelet agent (the use of pretreatment is not recommended by current guidelines when the second antiplatelet is prasugrel).

Further studies may shed some light on issues including the pretreatment with ticagrelor in NSTE-ACS (similar to what was performed in the ACCOAST study for prasugrel); maximal tolerable time until angiography is performed when the non-pretreatment strategy is chosen; subgroups with a favorable risk-benefit balance for the pretreatment (e.g. patients with a low bleeding risk according to validated scores and high probability of obstructive disease before angiography).
